# Prediction model for an early revision for dislocation after primary total hip arthroplasty

**DOI:** 10.1371/journal.pone.0274384

**Published:** 2022-09-09

**Authors:** Oskari Pakarinen, Mari Karsikas, Aleksi Reito, Olli Lainiala, Perttu Neuvonen, Antti Eskelinen

**Affiliations:** 1 Coxa Hospital for Joint Replacement, Tampere, Finland; 2 Faculty of Medicine and Health Technology, University of Tampere, Tampere, Finland; 3 Department of Orthopaedics and Traumatology, Tampere University Hospital, Tampere, Finland; 4 Department of Radiology, Tampere University Hospital, Tampere, Finland; Assiut University Faculty of Medicine, EGYPT

## Abstract

Dislocation is one of the most common complications after primary total hip arthroplasty (THA). Several patient-related risk factors for dislocation have been reported in the previous literature, but only few prediction models for dislocation have been made. Our aim was to build a prediction model for an early (within the first 2 years) revision for dislocation after primary THA using two different statistical methods. The study data constituted of 37 pre- or perioperative variables and postoperative follow-up data of 16 454 primary THAs performed at our institution in 2008–2021. Model I was a traditional logistic regression model and Model II was based on the elastic net method that utilizes machine learning. The models’ overall performance was measured using the pseudo R^2^ values. The discrimination of the models was measured using C-index in Model I and Area Under the Curve (AUC) in Model II. Calibration curves were made for both models. At 2 years postoperatively, 95 hips (0.6% prevalence) had been revised for dislocation. The pseudo R^2^ values were 0.04 in Model I and 0.02 in Model II indicating low predictive capability in both models. The C-index in Model I was 0.67 and the AUC in Model II was 0.73 indicating modest discrimination. The prediction of an early revision for dislocation after primary THA is difficult even in a large cohort of patients with detailed data available because of the reasonably low prevalence and multifactorial nature of dislocation. Therefore, the risk of dislocation should be kept in mind in every primary THA, whether the patient has predisposing factors for dislocation or not. Further, when conducting a prediction model, sophisticated methods that utilize machine learning may not necessarily offer significant advantage over traditional statistical methods in clinical setup.

## Introduction

In recent studies, the prevalence of dislocation after primary Total Hip Arthroplasty (THA) has been 0.4–4.1% [1–5], and it is one of the most common reasons for revision surgery in large THA registries [6–8]. The prevalence of dislocation is clearly higher (8.9–16%) after revision surgery [9–11]. Dislocation often becomes a recurrent problem that causes pain and limited functional ability for the patient and increased costs for the society, and is difficult to treat effectively [9,12]. Therefore, it is important to minimize the risk for dislocation already in the primary operation.

Several patient-related risk factors for dislocation have been reported, e.g. higher age, a previous spinolumbal fusion and higher BMI [13]. Patients operated for femoral neck fracture (FNF), rheumatoid arthritis or avascular necrosis of the femoral head have higher risk for dislocation compared with patients operated for primary osteoarthritis (OA) [14,15]. Some of the surgical and implant-related factors e.g. smaller femoral head size and smaller hospital volume have also been associated with an increased risk for dislocation [16,17]. While numerous studies have reported risk factors for dislocation, few studies have tried to predict dislocation or dislocation revision using correct methodology.

Prediction models can be useful tools for clinical decision making. Therefore, they are widely used in clinical medicine [18], and they have also been used to predict outcomes after THA [19–21]. Prediction models use predictor variables to estimate the probability or risk that the outcome is present (diagnostic model) or will occur within a certain time period (prognostic model) in an individual with a particular predictor profile [18]. In contrast to explanatory studies that aim to find a causal correlation between certain explanatory variable and the outcome variable, the aim in predictive studies is to build a prediction model with the best possible predictive capability for the outcome [22]. These two concepts are often confused in orthopaedic research [23]. Reliable preoperative risk prediction model would be very valuable because it would help in targeting the anti-dislocation devices (e.g. as dual-mobility cups or constrained liners) for the patients that are in high risk for recurrent dislocations.

The aim of this study was to build and to assess the performance of two prognostic prediction models for an early (within the first two years) revision due to dislocation. We aimed to reach this goal by using two different methodological approaches (logistic regression and elastic net) to assess the data in the joint replacement database at our institution.

## Materials and methods

### Source of data, participants and sample size

The data was collected from the electronic datalake of our high-volume academic tertiary joint replacement hospital. The same data were used in both prediction models. More specific patient information has been recorded to our institution’s database since 2008. Therefore, we included primary THAs performed at our hospital between 1.1.2008 and 30.8.2021 that either end up to revision for dislocation during the follow-up, or did not end up to revision for any reason during the follow-up (n = 16 777). The primary THA patients that were revised for other reason than dislocation during the follow-up (n = 920) were not included in the study. We excluded patients that received a constrained liner in the primary operation (n = 323). Primary operations where a dual-mobility cup was used (n = 134) were included because the indications of dual-mobility cup in primary THA do not differ significantly from conventional implants, while the indications for constrained liner are more restricted. The final data included 16 454 primary THAs. The follow-up ended in the day of death, revision surgery or 30.8.2021 (date of data collection), whichever came first. We used revision for dislocation as the endpoint instead of mere dislocation, because the dislocations treated with closed reduction in the local emergency department are not automatically recorded in our database, and marked proportion of one-time dislocators do not require revision surgery [24]. Revision was defined as a new surgical procedure including partial or complete removal or exchange of any THA component.

### Outcome

The main outcome in both prediction models was revision for dislocation within the first 2 years after primary THA.

### Statistical analyses and predictors–Model I

The first prediction model was a logistic regression model. The predictors were chosen from variables that have been associated with dislocation in the previous literature or were thought to possibly affect the risk for dislocation in some causal pathway. The variable selection was done according to the recommendations by Heinze et al. [25]. The main literature source was a recent systematic review and meta-analysis related to the risk factors for dislocation [13]. Age, sex, American Society of Anaesthesiologists (ASA) score, Carlson Comorbidity Index (CCI), body mass index (BMI), primary reason for operation (primary osteoarthritis / hip fracture / avascular necrosis of the femoral head / rheumatoid arthritis / tumor / other), history of psychiatric or neurological diseases, serum creatinine level, serum mean corpuscular volume (MCV), and use of Parkinson’s disease medicine or antiepileptic medicines within the last 365 days were included from patient-related factors. Femoral and acetabular fixation method (cemented/uncemented) and femoral head size were included in the model from the surgical or implant-related factors. A redundancy analysis based on linear regression was performed to detect potential collinearity between the predictors before analyzing the actual prediction model. Missing data was imputed using multiple imputation for final model estimation. For calibration single imputation was used. The overall performance (i.e. the predictive capability) of the final prediction model was assessed by calculating the Nagelkerke´s R^2^ value for the model. The R^2^ value (ranging from 0 to 1) represents the proportion of the variance in the outcome variable that is explained by the prediction variables of the model. The discrimination of the model was assessed by calculating the C-index. A 0.5 C-index represents random concordance, and a model with a 1.0 C-index would predict every outcome in the data correctly. Restricted cubic splines with three knots were used for the continuous variables (age, BMI, serum creatinine and MCV) [26]. The relative importance of the single prediction variables in the final model was assessed by calculating the chi-squared regression coefficients with p values for the predictors based on the Wald statistic.

### Statistical analyses and predictors–Model II

**Fig 1:**
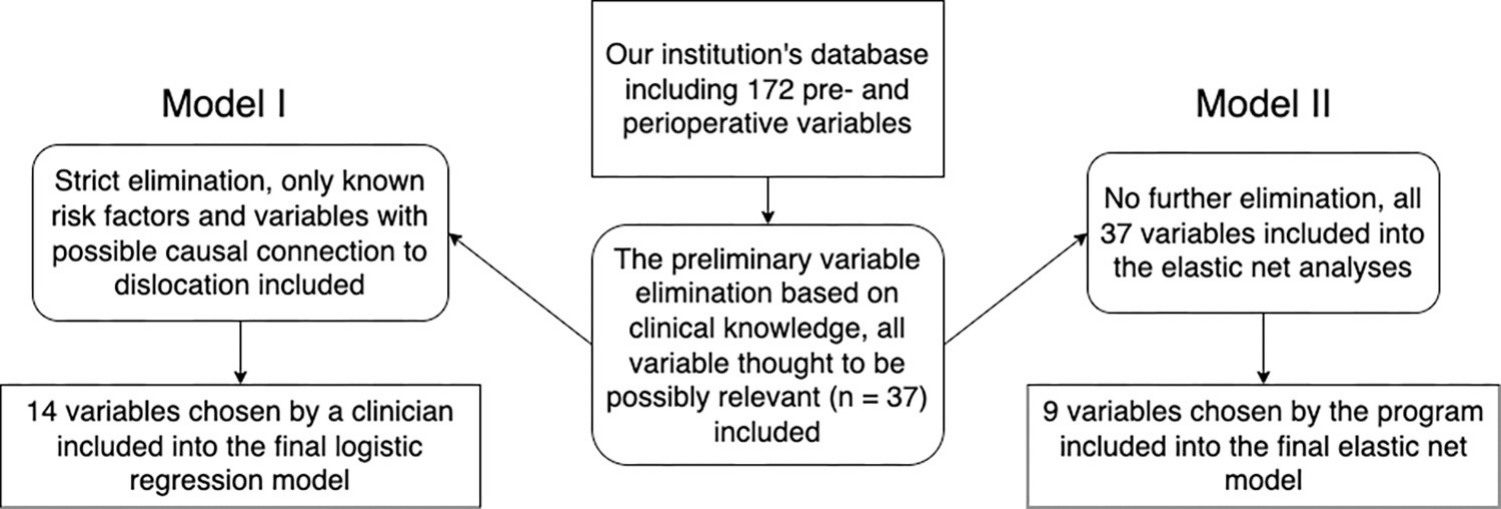
The flow chart summarizing the predictor variable choice for Model I and Model II.

The second prediction model was an elastic net model. The elastic net method is a machine learning method based on generalized linear regression. Elastic net regularization method systematically searches the optimal combination of predictors by using so called regularization penalties of the loss functions. It computationally removes the weak and highly correlated predictors and chooses the best combination of variables to be involved in the final model [27]. The elastic net method has been shown to be a useful tool in total joint arthroplasty -related prediction studies [28]. In this analysis, highest area under the curve (AUC) was the target. Similarly to the C-index, the AUC measures the discriminative ability of the model. The pseudo R^2^ value (comparable with the R^2^ value in Model I) was calculated for the elastic net model. All variables found in our database that were thought to be possibly relevant were included. In addition to all the variables used in the Model I, the following variables were involved in the elastic net analyses: Preoperative hip range of motions (extension, flexion, abduction, adduction, internal and external rotation), Trendelenburg sign, history of psychosis or previous injury, diagnosis of osteoporosis, anemia, dementia, diabetes or hemiplegia, smoking status, use of corticosteroids, muscle relaxants, beta blockers, antidiabetics or drugs for gastrointestinal disorders within the last 365 days, used surgical approach and whether the surgery was bilateral operation or a teaching operation. In this model, age and BMI were analyzed as categorical variables based on cut values (0–50, 50–65, 65–75, 75+ for age; 0–25, 25–30, 30–35, 35–40, 40+ for BMI). The missing data in categorical variables were handled by creating a new category for missing values within the variable. The relative importance of single prediction variables was not analyzed in this model. The flow chart summarizing the variable choice in both models is presented in Fig 1.

All the analyses were performed using R statistical software (R Centre for Statistical Computing, Vienna, Austria). For Model I rms package was used, and for Model II glmnet package was used.

### Ethics

According to the Finnish research legislation, the review of the ethics committee is not required for the research of registry data [29]. According to the Finnish National Board on Research integrity (https://tenk.fi/en/advice-and-materials/guidelines-ethical-review-human-sciences, Chapter 4.2 RESEARCH DESIGN ELEMENTS REQUIRING ETHICAL REVIEW, first subheading “More specific information on the above elements requiring ethical review”, second paragraph): “The review of the ethics committee is not required for the research of public and published data, registry and documentary data and archive data." Therefore, the Ethics Committee of Tampere University Hospital, which includes our department, has waived ethical evaluation of all register-based studies, in which the participants are not contacted [30]. Permission to use our hospital’s database was obtained from Institutional Review Board at our hospital. Informed consent from patients was not needed as we used retrospective register data and therefore the participants were not contacted.

## Results

At 2 years postoperatively, 95 hips (0.6% prevalence) had been revised for dislocation. During the whole follow-up period, 158 hips had been revised because of dislocation (1.0% prevalence). The median follow-up in the whole study population was 3.9 years (IQR 1.8–7.2). The patient demographics are presented in Table 1.

**Table 1:** Patient demographics.

	Dislocation revision	No dislocation revision
Number of patients (n)	158	16296
Follow-up (Years, median, IQR)	1.4 (0.2–3.0)	4.0 (1.8–7.2)
Age (Years, mean, SD)	68 (10)	67 (11)
BMI* (Mean, SD)	29 (5.0)	28 (5.2)
Sex (n, %)		
Male	70 (44)	6973 (43)
Female	88 (56)	9323 (57)
Primary reason for operation (n, %)		
Primary osteoarthritis	126 (80)	13832 (85)
Femoral neck fracture	8 (5.1)	541 (3.3)
Developmental dysplasia of the hip	3 (1.9)	335 (2.1)
Avascular necrosis of the femoral head	7 (4.4)	340 (2.1)
Other reason	14 (8.9)	1212 (7.4)
Data missing	0 (0.0)	36 (0.2)
ASA score (n, %)		
1	14 (8.8)	2372 (15)
2	69 (44)	7446 (46)
3	67 (42)	5484 (34)
≥4	2 (1.3)	344 (2.1)
Data missing	6 (3.8)	650 (4.0)
Psychiatric or neurological disease (n, %)		
Yes	43 (27)	2971 (18)
No	108 (68)	12733 (78)
Data missing	7 (4.4)	592 (3.6)
Approach (n, %)		
Posterior	143 (91)	14 653 (90)
Anterior	7 (4.4)	432 (2.7)
Anterolateral	1 (0.6)	17 (0.1)
Data missing	7 (4.4)	1194 (7.3)
Acetabular fixation (n, %)		
Cemented	15 (9.5)	2015 (12)
Uncemented	143 (91)	14 252 (88)
Data missing	0 (0.0)	29 (0.2)
Femoral fixation (n, %)		
Cemented	93 (59)	7851 (48)
Uncemented	65 (41)	8437 (52)
Data missing	0 (0.0)	8 (0.0)
Femoral head size (n, %)		
22 mm	0 (0.0)	3 (0.0)
28 mm	2 (1.3)	272 (1.7)
32 mm	28 (18)	2628 (16)
35 mm	0 (0.0)	30 (1.8)
36 mm	113 (72)	11632 (71)
≥ 40 mm	4 (2.5)	516 (3.2)
Dual-mobility cup	1 (0.6)	133 (0.8)
Data missing	10 (6.3)	1082 (6.6)

BMI = Body Mass Index, ASA score = American Society of Anesthesiologists score.

*BMI coverage in the data: 96%.

**Fig 2:**
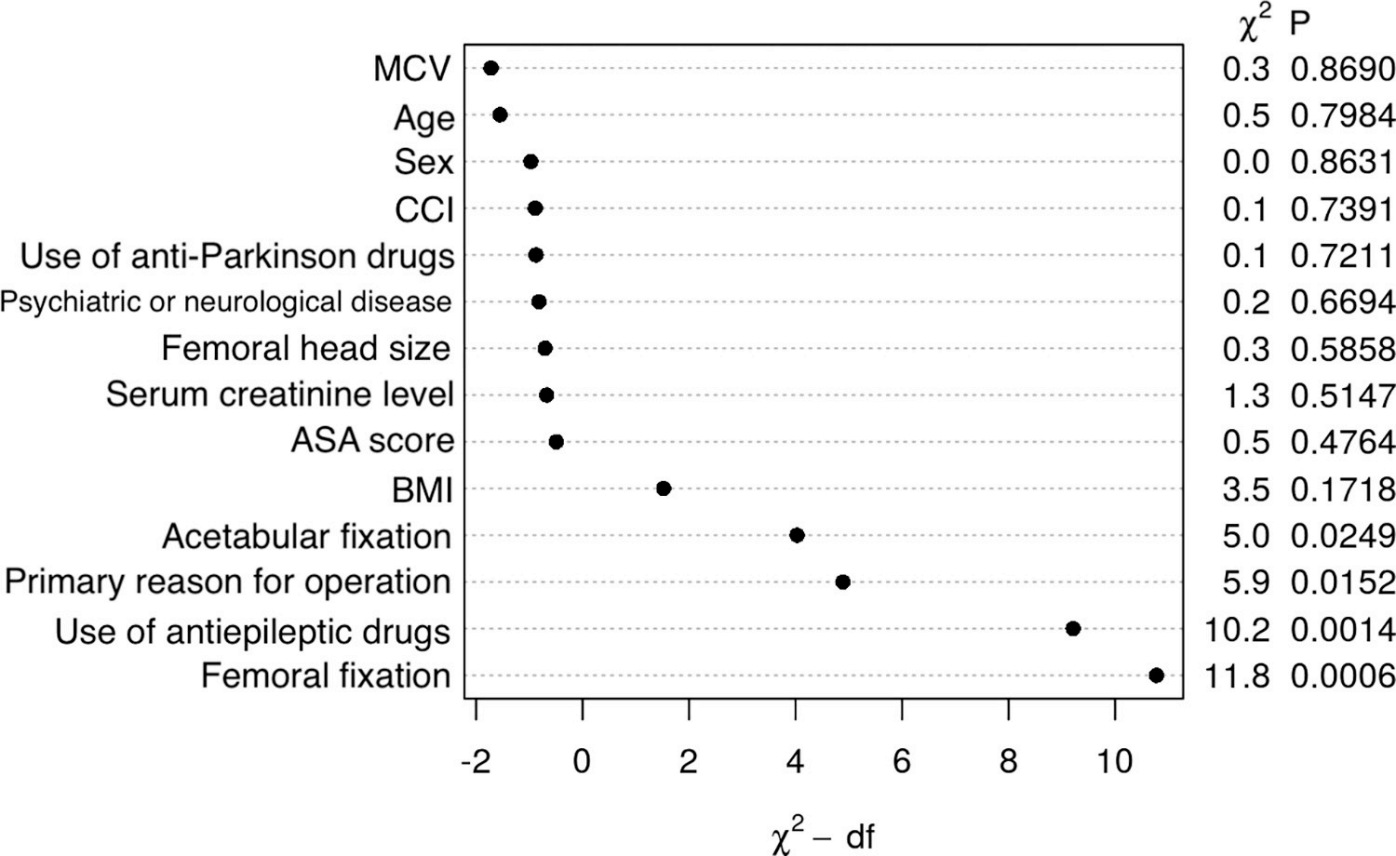
The Chi-squared regression coefficients with p values for the predictors in the Model I (logistic regression model). Femoral fixation was the most important and MCV the least important predictor in this model. MCV = Mean corpuscular volume, CCI = Carlson Comorbidity Index, ASA score = American Society of Anesthesiologists score, BMI = Body mass index.

In the Model I (logistic regression prediction model), all of the final 14 prediction variables chosen by a clinician were included based on the results of the redundancy analysis (Supplementary 1). The C-index of the prediction model was 0.67. The R^2^ value was only 0.04 indicating low predictive capability. Femoral fixation, use of antiepileptic medicine and primary reason for operation were the most important predictors for an early dislocation revision (Fig 2). In the Model II (elastic net prediction model), the program chose nine variables to be included into the final model: preoperative hip internal rotation, avascular necrosis of the femoral head as the indication for surgery, preoperative anemia, femoral fixation type, BMI cut value of 30–35, the history of psychosis and the use of antiepileptic medicines, muscle relaxants and diabetes drugs. An AUC of 0.73 (95% CI 0.67–0.78) was achieved with this combination of variables. The R^2^ value (0.02) was low also in this model.

**Fig 3:**
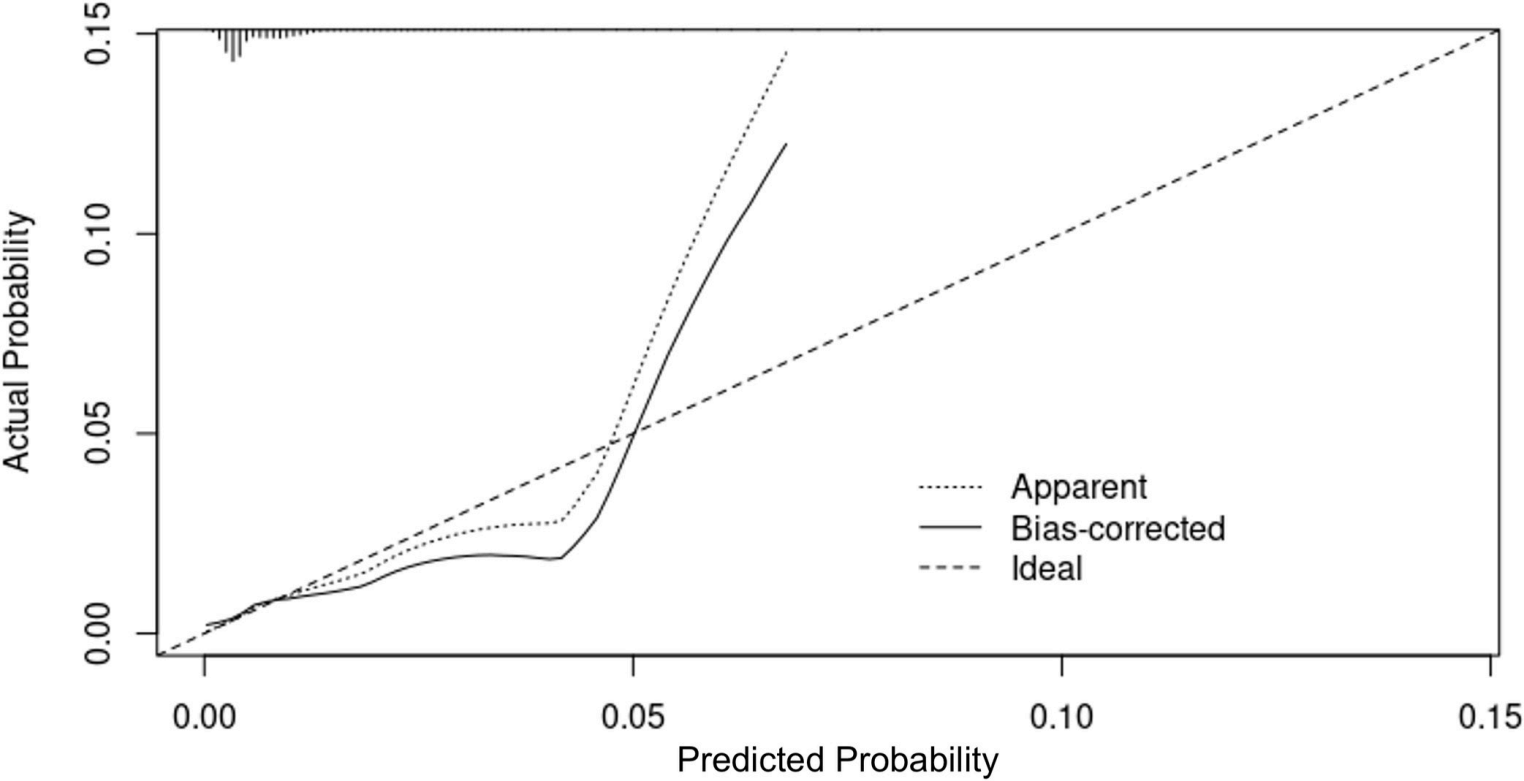
The calibration curve for the Model I (logistic regression model). On the x-axis are the predicted probabilities and on the y-axis the observed probabilities. A perfectly calibrated model would follow the straight dashed line referred as “ideal” in the picture. On average, the Model I overestimated the probability of revision for dislocation in patients that had low probability of this outcome, and underestimated the probability of revision for dislocation in patients that had the highest probability of this outcome.

**Fig 4:**
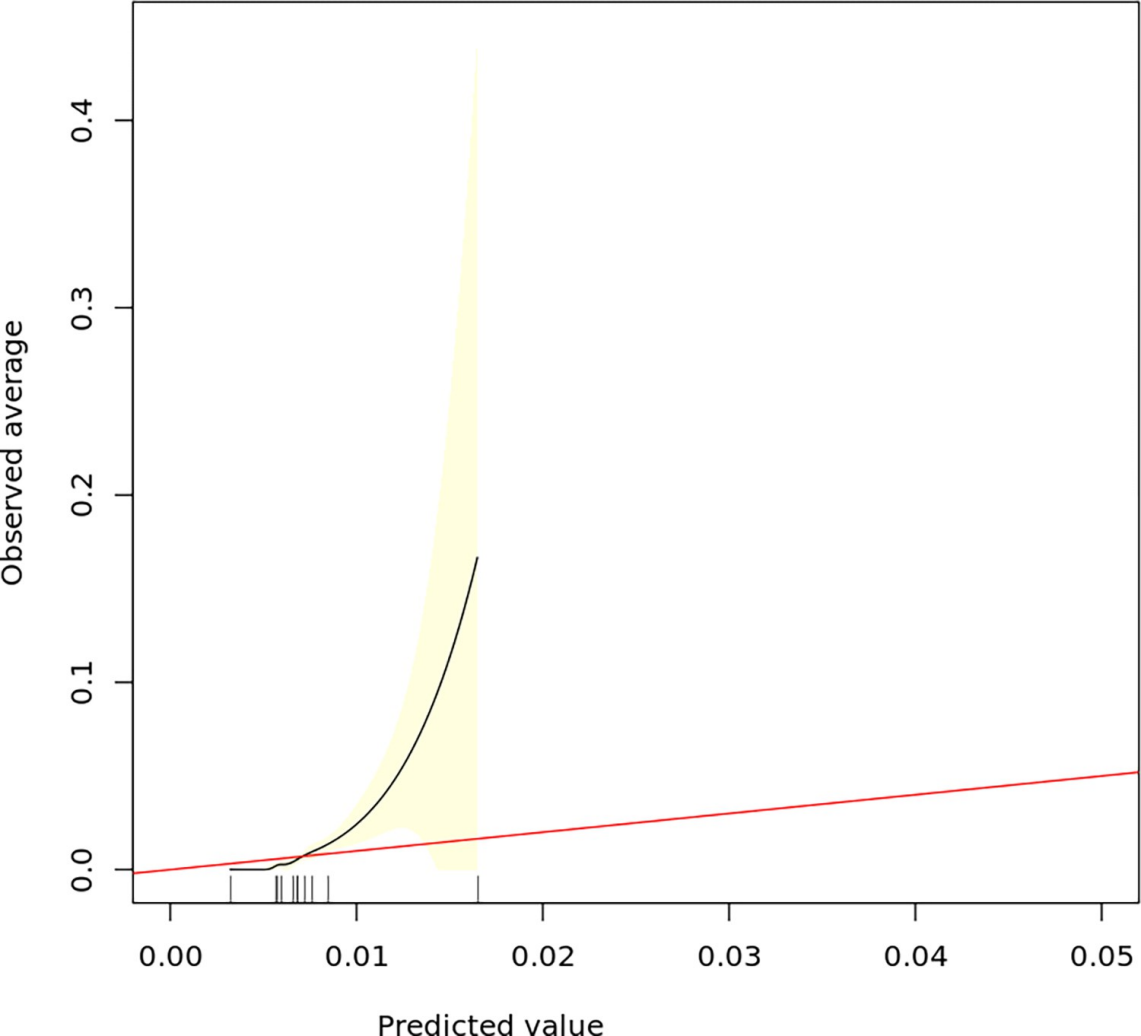
The calibration curve for the Model II (elastic net). On the x-axis are the predicted probabilities and on the y-axis are the observed probabilities. The predicted probability (black curve) moves farther from the true probability (red line) as the predicted value increases, indicating that the model underestimates the probability of dislocation for the patients having the highest probability of this outcome.

**Fig 5:**
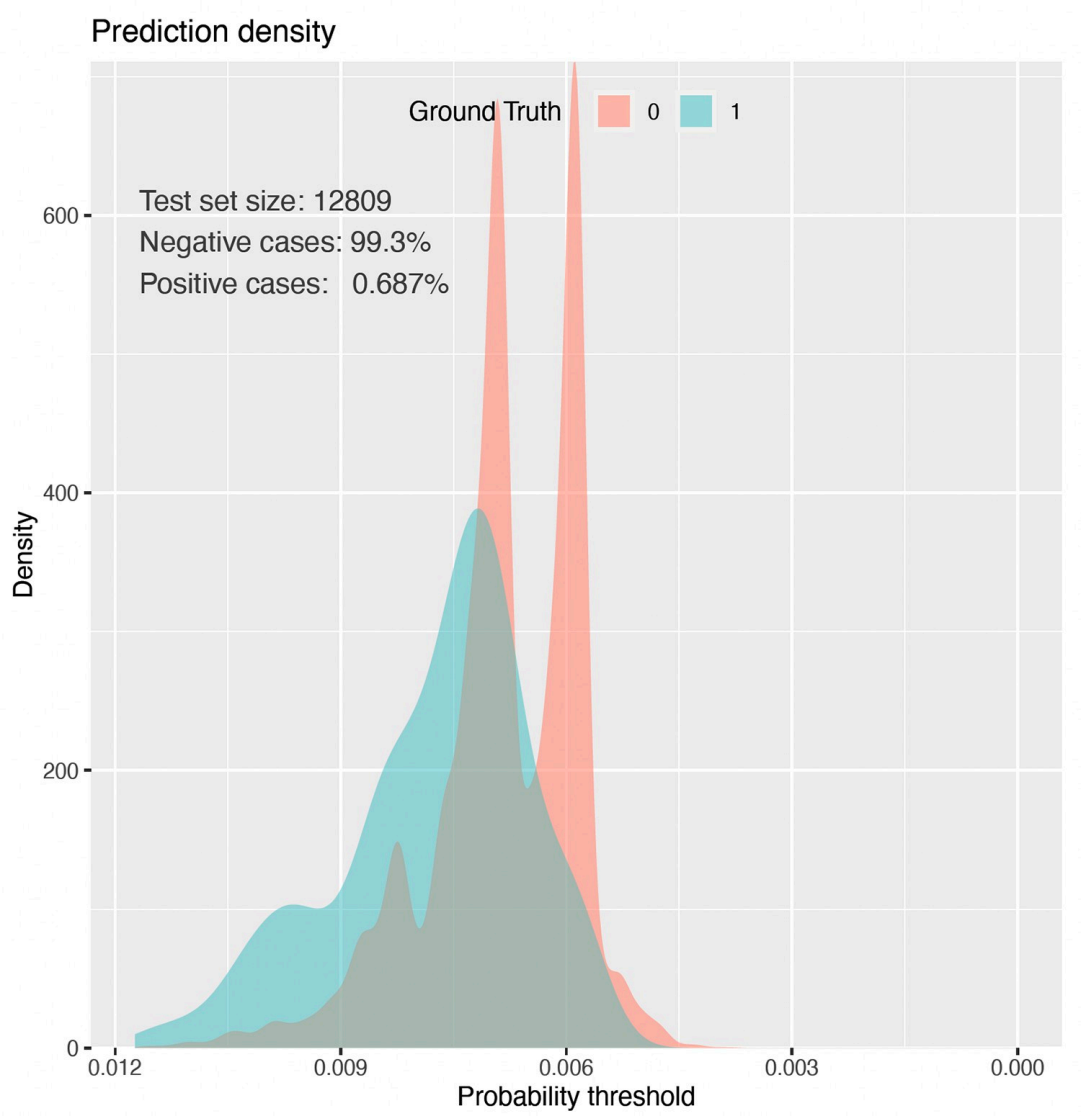
The prediction density graph for the Model II (elastic net). This graph presents how well the model discriminates the patients revised for dislocation (blue) from patients not revised for dislocation (red). In an ideal model the blue and red parts would be completely separated.

The calibration graph for Model I is presented in Fig 3, and the calibration graph for Model II is presented in Fig 4. The prediction density graph for Model II is presented in Fig 5.

## Discussion

We found that the prediction of dislocation revision within the first 2 years after primary operation is very difficult even when a large and specific patient- and operation-related data is available for the analyses. The predictive capability of the logistic regression model (R^2^ 0.04, C-index 0.67) and the elastic net model (R^2^ 0.02, AUC 0.73) were approximately in the same level.

Even though multiple THA-related prediction models have been published, few of them have dealt with dislocation or revision for dislocation. Venäläinen et al. built a prediction model for an early revision for dislocation after primary THA based on pre- and perioperative data from 25 919 operations from the Finnish Arthroplasty Register. Their model reached an AUC of 0.64 for revision for dislocation within the first 6 months after primary THA [31]. In their study revision for dislocation was the most difficult endpoint to predict (other endpoints were revision for infection, revision for periprosthetic femoral fracture and death). Our results are in line with Venäläinen et al., since even though the discrimination in both of our models was higher compared with the AUC in their study, the overall predictive ability in our models was low. Rouzrokh et al. predicted dislocation after primary THA based on the cup position measured from 92 584 postoperative radiographs from 13 970 operations using convolutional neural networks [32]. Their model reached 89% sensitivity, 49% specificity, and an AUC of 0.77. According to their results, dislocation can be predicted moderately based on the cup position. However, the cup position is not a factor that can be assessed preoperatively, and therefore it is not helpful when judging patient’s individual risk for dislocation before the primary THA. Reliable preoperative risk prediction would be very valuable for the clinicians, as it would aid in targeting the anti-dislocation devices, such as dual-mobility cups or constrained liners, for the patients in high risk for recurrent dislocations. As these devices introduce the risk of wear and other mechanical complications in the long-term, they should be reserved for those patients who benefit the most from them.

Machine learning (ML) is a hypernym for collection of techniques that allow the computers to undertake difficult tasks with complex algorithms. ML-based methods can be especially beneficial when conducting prediction models with extensive data. In recent years, the role of ML is emphasized in medical research [33]. The elastic net is one example of ML-based methodology. There are recent examples where ML-based prediction models reach high predictive capability of postoperative falls [34] and medical complications [35] after THA. However, the general superiority of ML over more traditional statistical methods has yet not been proved. A recent systematic review assessed the discrimination of clinical prediction models for binary outcomes using either logistic regression or ML [36]. In that study a difference could not been found in the AUC values between logistic regression and ML models (0.00 difference, 95% CI -0.18–0.18) when studies having low risk of bias were analyzed. In our study the AUC was higher in the elastic net model compared with the C-index in logistic regression model but the R^2^ value was lower.

In our study, femoral fixation was the most important predictor for dislocation revision in the Model I (logistic regression model). Cemented stems are favored in old and fragile patients who tend to have higher risk of falling and fractures [37,38]. It seems evident that in our study cemented femoral fixation has been used for more dislocation-prone patients on average, because it is very unlikely that the choice of femoral fixation method would significantly affect the risk for dislocation. The use of antiepileptic medicines was the second most important predictor in the logistic regression model. The diagnosis of epilepsy has not been associated with dislocation in previous studies, but in theory an epileptic seizure could predispose to dislocation. Moreover, the Anatomical Therapeutic Chemical code 03 includes also benzodiazepine derivates that are associated with increased risk of injurious falls [39]. Benzodiazepines are also used in the treatment of severe alcohol withdrawal [40], and alcohol abuse has been associated with increased risk for dislocation [41]. Nonetheless, it is not possible to assess causal connections in this study design. The third most important predictor in the logistic region model was the primary reason for operation. This was expectable since patients operated for femoral neck fracture, avascular necrosis of the femoral head, developmental dysplasia of the hip and rheumatoid arthritis have been associated with an increased risk for dislocation compared with patients operated for primary osteoarthritis [14,15,42–44]. The femoral fixation, use of antiepileptic medicines and avascular necrosis of the femoral head as the indication for primary THA were also chosen by the elastic net program to be included in the Model II. However, because the combined predictive capability of these variables was limited in our study, strong conclusions cannot be made based on our results.

We acknowledge a few limitations in the current study. We could only predict revision for dislocation, because the dislocations that are treated with closed reduction in the local emergency department are not automatically recorded in our hospitals’ database. Dislocation is more stochastic event compared with revision for dislocation, and therefore would be better endpoint for prediction model. For instance, surgeon’s evaluation and patient’s opinion and compliance after dislocation(s) affect the probability of revision for dislocation but these factors cannot be evaluated in the preoperatively gathered patient information. We could only compare the dislocations within 2 years after primary THA because the variability in the follow-up lengths in our data would have made long-term comparison problematic. Because an early revision for dislocation is quite rare event, it is challenging to build an effective prediction model even with a large dataset with plenty of relevant predictor variables. In our analyses we could not validate the prediction models by dividing the data to teaching cohort and test cohort because of the low event rate.

## Conclusions

The prediction of an early revision for dislocation after primary THA is difficult even in a large cohort of patients with specific patient- and operation-related data available because of reasonably low prevalence and multifactorial nature of dislocation, and due the fact that not all dislocation lead to revision. The risk of dislocation should be kept in mind in every primary THA, whether the patient has predisposing factors for dislocation or not. Further, when conducting a prediction model, sophisticated methods that utilize machine learning may not necessarily offer significant advantage over traditional statistical methods in clinical setup.

## Supporting information

S1 Table The redundancy analysis for the logistic regression prediction model. The R^2^ value for a single predictor variable indicates the proportion of the variance of that predictor variable that is explained by other prediction variables of the Model I. In all variables the R^2^ value was well below 0.8 that was defined as threshold value. Therefore, none of the variables were excluded from the final prediction model. ASA score = American Society of Anesthesiologists score, CCI = Carlson Comorbidity Index, BMI = Body mass index, MCV = Mean corpuscular volume.
(DOCX)
