# Supplementary material for: Prediction model for an early revision for dislocation after primary total hip arthroplasty
Source: PLoS One. 2022 Sep 9;17(9):e0274384. doi: 10.1371/journal.pone.0274384 (PMC9462822; doi:10.1371/journal.pone.0274384)
Supplement: S1 Table — The R2 value for a single predictor variable indicates the proportion of the variance of that predictor variable that is explained by other prediction variables of the Model I. In all variables the R2 value was well below 0.8 that was defined as threshold value. Therefore, none of the variables were excluded from the final prediction model. ASA score = American Society of Anesthesiologists score, CCI = Carlson Comorbidity Index, BMI = Body mass index, MCV = Mean corpuscular volume. (DOCX) [file pone.0274384.s001.docx]

Supplementary 1. The redundancy analysis for the logistic regression prediction model. The R^2^ value for a single predictor variable indicates the proportion of the variance of that predictor variable that is explained by other prediction variables of the Model I. In all variables the R^2^ value was well below 0.8 that was defined as threshold value. Therefore, none of the variables were excluded from the final prediction model.

| Prediction variable | R2 value |
| --- | --- |
| Age | 0.48 |
| Sex | 0.16 |
| ASA score | 0.38 |
| CCI | 0.17 |
| BMI | 0.13 |
| Primary reason for operation | 0.09 |
| Psychiatric or neurological disease | 0.08 |
| Serum creatinine level | 0.13 |
| MCV | 0.02 |
| Use of anti-Parkinson drugs | 0.02 |
| Use of antiepileptic drugs | 0.03 |
| Femoral fixation | 0.33 |
| Acetabular fixation | 0.14 |
| Femoral head size | 0.10 |

ASA score = American Society of Anesthesiologists score, CCI = Carlson Comorbidity Index, BMI = Body mass index, MCV = Mean corpuscular volume.
